# Sago-Type Palms Were an Important Plant Food Prior to Rice in Southern Subtropical China

**DOI:** 10.1371/journal.pone.0063148

**Published:** 2013-05-08

**Authors:** Xiaoyan Yang, Huw J. Barton, Zhiwei Wan, Quan Li, Zhikun Ma, Mingqi Li, Dan Zhang, Jun Wei

**Affiliations:** 1 Institute of Geographical Sciences and Natural Resources Research, Chinese Academy of Sciences, Beijing, China; 2 School of Archaeology and Ancient History, University of Leicester, Leicester, United Kingdom; 3 Graduate University of Chinese Academy of Sciences, Beijing, China; 4 Guangdong Provincial Museum, Guangzhou, China; University of Florence, Italy

## Abstract

Poor preservation of plant macroremains in the acid soils of southern subtropical China has hampered understanding of prehistoric diets in the region and of the spread of domesticated rice southwards from the Yangtze River region. According to records in ancient books and archaeological discoveries from historical sites, it is presumed that roots and tubers were the staple plant foods in this region before rice agriculture was widely practiced. But no direct evidences provided to test the hypothesis. Here we present evidence from starch and phytolith analyses of samples obtained during systematic excavations at the site of Xincun on the southern coast of China, demonstrating that during 3,350–2,470 aBC humans exploited sago palms, bananas, freshwater roots and tubers, fern roots, acorns, Job's-tears as well as wild rice. A dominance of starches and phytoliths from palms suggest that the sago-type palms were an important plant food prior to the rice in south subtropical China. We also believe that because of their reliance on a wide range of starch-rich plant foods, the transition towards labour intensive rice agriculture was a slow process.

## Introduction

The orthodox view of the spread of agriculture in southern China and southeast Asia is that Neolithic farmers spread south from mainland China on to Taiwan and then into island Southeast Asia. In doing so, they are argued to have spread a cultural package of domesticated rice, pigs, forms of pottery, along with Austronesian languages. The initial cultural migration from southern China is thought to have occurred sometime around or prior to 5,000 years ago, ultimately terminating in the colonization of remote Oceania by 1,200 AD [1]–[3].This model is built upon evidence from archaeological, as well as linguistic and genetic data [1]–[5], but importantly, from many sites used in the model, archaeobotanical data is lacking. Because of poor organic preservation at many of these southern subtropical sites, we know very little about the typical plant diets south of the Yangtze River Basin. As a result archaeologists have been forced to rely on data from historic sites and written records to infer the nature of subsistence in this region [3].

According to the historic records it is generally presumed that roots and tubers were the likely staple plant foods during the prehistoric period [6]–[8]. This view has been supported by limited archaeological evidence of charred roots and tubers, although unidentified to species recovered from Zengpiyan cave, an early Holocene site in Guangxi, south China [9]. Xincun site, reported here, clarifies for the first time, the plant use traditions that dominated southern subtropical China and by reference other parts of Southeast Asia, before the spread of rice cultivation.

**Figure 1:**
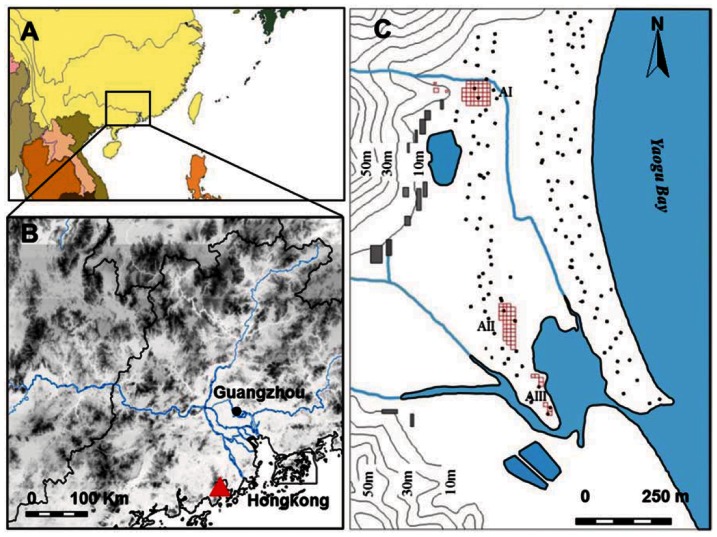
The Xincun Site on the southern coast of China. Location of the study region (A), the site is indicated by the red triangle (B), and geomorphological features of the Xincun site (C). Red grids mark excavation area (AI-III), stippling shows coastal sand dunes.

The Xincun site (112°59′E, 21°54′N) is located at 7.0 m a.s.l. along the crest of a coastal sand dune, approximately 180 km southwest of Guangzhou City (Figure 1). The site was excavated from July 2008 through April 2009, ahead of a major industrial development in the area, uncovering ∼8,000 m^2^ a series of village occupations. This settlement was located near a remnant of an older lagoon into which freshwater streams flew from surrounding hills (Figure 1). During the excavations six late Neolithic layers were identified alternating with sandy layers, 15–40 cm thick, indicating periods of site abandonment. Ten AMS dates derived from charcoal and soot from the exterior faces of pottery fragments placed the site occupations between *ca.*3,500 aBC and 2,470 aBC (Table 1). Features included living surfaces, postholes, pits of various types and hearths were systematically uncovered. The material culture is characterized by sand tempered pottery, stone tools including grinding slabs and pestles, grooved pebbles, net weights, and pierced pebble sinkers for fishing.

**Table 1:** AMS radiocarbon dates from occupation layers, Xincun site.

Lab No.	Sample	Field No.	ALS§	^14^C (BP)	Calibrated 2σ (95.4%)
BA090573	charcoal	TXTN66W14H52:SP1	3	4425±35	3330BC (20.1%) 3210BC3180BC (2.1%) 3150BC3130BC (73.1%) 2920BC
BA090574	charcoal	TXTN67W12C36:SP1	1	4060±40	2860BC (10.4%) 2810BC2750BC (2.4%) 2720BC2700BC (82.6%) 2470BC
BA090575	charcoal	TXTN68W14:SP1	4	4480±35	3350BC (89.1%) 3080BC3070BC (6.3%) 3020BC
BA090576	charcoal	TXTN69W15C73:SP1	3	4490±35	3350BC (92.0%) 3080BC3060BC (3.4%) 3030BC
BA090578	charcoal	TXTN68W15C16:SP1	1	4220±40	2910BC (36.8%) 2830BC2820BC (58.6%) 2670BC
BA090579	soot	TXTN66W15H51:SP1	4	5050±40	3960BC (93.7%) 3760BC3740BC (1.7%) 3710BC
BA090580	charcoal	TXTN66W16:SP1	2	4550±50	3500BC (4.3%) 3450BC3380BC (91.1%) 3090BC
BA090581	charcoal	TXTN67W16C56:SP1	2	4440±40	3340BC (32.6%) 3210BC3190BC (6.7%) 3150BC3140BC (56.2%) 2920BC
BA090583	soot	TXTN68W10:SP1	1	3830±45	2460BC (90.0%) 2190BC2180BC (5.4%) 2140BC
BA090584	soot	TXTN13W5:SP1	4	4450±35	3340BC (91.7%) 3000BC2990BC (3.7%) 2930BC

Note:

§ ALS is Ancient Living Surface. Half life of carbon is 5568 and BP is before 1950. Tree ring curve for calibaration is IntCalo4(1), and progremee for calibartion is OxCal v3.10(2).

• Reimer PJ, MGL Baillie, E Bard, A Bayliss, JW Beck, C Bertrand, PG Blackwell, CE Buck, G Burr, KB Cutler, PE Damon, RL Edwards, RG Fairbanks, M Friedrich, TP Guilderson, KA Hughen, B Kromer, FG McCormac, S Manning, C Bronk Ramsey, RW Reimer, S Remmels, JR Southon, M Stuivers, S Talamo, FW Taylor, J van der Plicht, and CE Weyhenmeyer. 2004 *Radiocarbon* 46:1029–1058.

• Christopher Bronk Ramsey 2005, www.rlaha.ox.ac.uk/orau/oxcal.html.

## Materials

**Figure 2:**
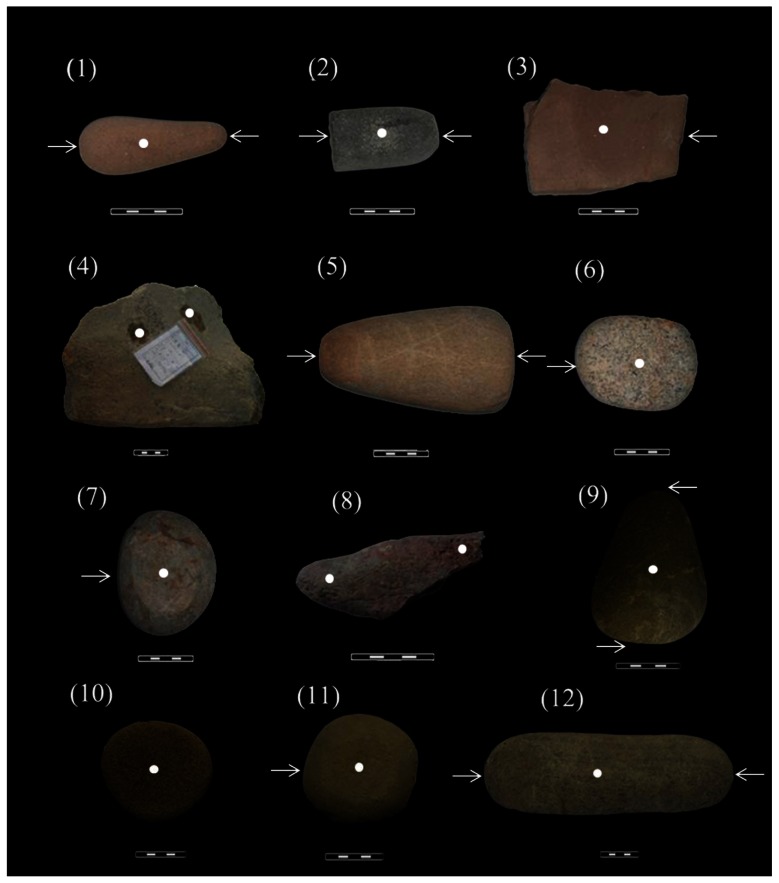
Stone tools examined for starch residues (1#–8#) and phytoliths (9#–12#). White dots and arrows indicate sampling locations. Scale bar: 5 cm. 1-*n* indicate sample numbers used in Table 1.

Flotation work was carried out at the site during the excavation but no macrofossils were recovered. However, samples for starch and phytolith analyses were systematically obtained from objects recovered in three ancient living surfaces, No. 1, 3 and 5, from the upper layer to the lower layer. A total of 12 typical stone tools used as mullers, ground stones, pounders or pestles (Figure 2), of which eight were analysed for starches and four for phytoliths (Table 2). Two sediment samples immediately beneath living surface 2 were examined as a control for the presence of starch.

**Table 2:** Types and amounts of starches and phytolith on milling stones from the Xincun Site.

Tool #a)	Field number	Tool type	Starch typeb)									
			a	b	c	d	e	f	g	h	i	Total
1	TN69W13C11:14	Pestle	3	3	1				12		3	21
2	TN69W14C27:15	Muller/Pounder		10					1		5	17
3	TN69W13C11:6	Ground stone	16	4					23		19	63
4	TN68W13C18:17	Ground stone	2				18					21
5	TN67W13C30:1	Pestle			1	1					1	3
6	TN69W15C57:7	Muller/Pounder	2						13			19
7	TN68W14C85:5	Muller/Pounder	4			3	2				2	11
8	TN68W14C85:3	Pestle	58			13	9	38	4	5	178	305
Total			85	17	2	17	29	38	53	5	208	454

Tool #a)	Field number	Tool type	Phytolith typec)									
			j	k	l	m	n	o	p	q	r	Total
9	TN69W14C27:9	Muller/Pounder	103	8	1	5	12	17	3	93	5	247
10	TN67W13C30:43	Pestle	338	12	2	7	26	22	5	196	3	611
11	TN69W13C11:22	Pestle	289	8	3	4	14	20	14	184	1	537
12	TN67W15C66:10	Muller	364	6	1	0	18	24	10	130	2	555
Total			1094	34	7	16	70	83	32	603	11	1950

a) Stone tools 1–5, 9–11 from ancient living surface (ASL) 1; 6 &12 from ASL2; and 7 & 8 from ASL5, slightly earlier than 3,350-3,080a BC (BA090575).

b) Starch types. a, palms; b, banana (*Musa* sp.); c, lotus (cf. *Nelumbo nucifera*); d, Chinese arrowhead (*Sagittaria* sp.); e, water chestnut (cf. *Eleocharis dulcis*); f, fern (*Angiopteris* sp.); g, Job's-tear (*Coix* spp); h, acorn (*Quercus* sp.); i, starch granules damaged and/or unidentifiable.

c) Phytolith types.

j, globular echinate type from palms; k, polygonal cone from sedge; l, elongate of triangular prism from fern; m, sum of bulliform, two-peak glume and bilobate from *Oryza*; n, bulliform echinate and long saddle types from bamboo; o, broadleaved-tree phytolith; p, reed bulliform; q, sum of other types including conifer-tree phytolith, rondel from other grasses, etc. The detailed data showed in Figure 7. r, unidentifiable phytoliths.

## Methods

### Modern reference collections

Starch granule identification was based upon one-on-one comparisons between ancient starches and modern reference collections of more than 150 Asian species housed at the Institute of Geographic Sciences and Natural Resources Research (IGSNRR) at the Chinese Academy of Sciences, and 30 species housed at the Residue Lab of University of Leicester, UK, including 30 genera within the families of Alismataceae, Araceae, Arecaceae, Cycadaceae, Cyperaceae, Fagaceae, Fabaceae, Juglandaceae, Musaceae, Nymphaeaceae and Poaceae. All modern references were collected by authors from botanic gardens and field investigation. Comparative materials available from published studies were also consulted [10]–[14].

### Sampling and Extraction of starches

Stone tools selected for sampling were initially cleaned by brush to remove adhering dust from storage and then washed clean with ultra-pure water. Cavities on the surface of the tools were targeted for residue removal. We applied between 20–40 microlitres of ultra-pure water to areas of interest and left these to hydrate for 3–5 minutes. The wetted area was agitated with a metal pin to dislodge the sediment within the cavities. Finally a sample of this material was removed with a micropipette and transferred to a snap-cap vial for storage. For detailed of starch extraction, slide making and microscope observation please see Yang et al [14]. All necessary permits for the described field investigations were obtained from Guangdong Provincial Administration of Cultural Relics.

### Extraction of phytoliths

Recovery of residues for analysis followed that of sampling for starch. Eight samples of surface residues from used and non-used facets were treated with standard procedures of phytolith extraction [15]–[16]. Phytolith nomenclature and descriptions were consistent with International Code for Phytolith Nomenclature 1.0 [17] and Piperno [18].

## Results

In total of 454 starch granules and 1,950 phytoliths were recovered (Table 2). No starch granules were recovered from two sediment samples, demonstrating that the ancient starches were related to tool use.

**Figure 3:**
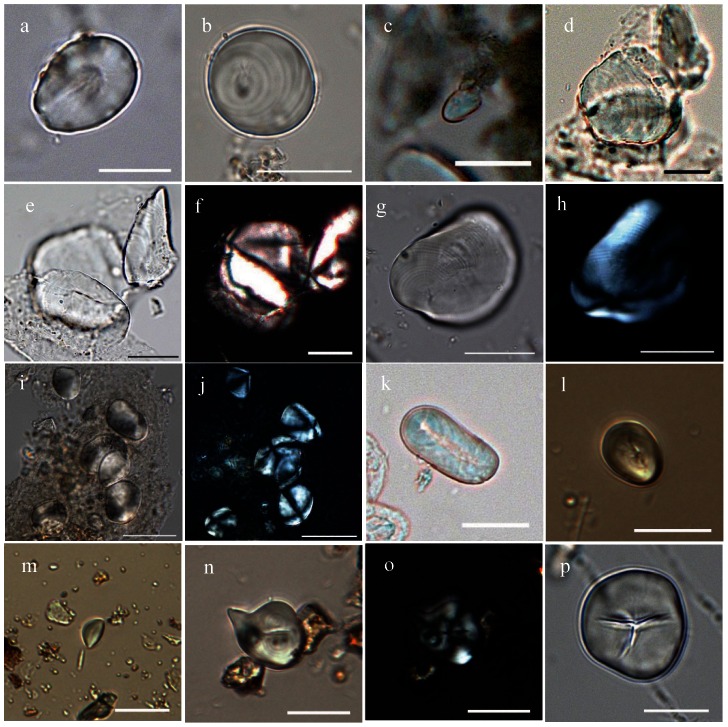
Ancient starches recovered from residues on the stone tools. a, *Caryota* sp., range, 12.1–25.8 µm; b, *Corypha* sp., range, 10.6–15.5 µm; c, possibly *Arenga* sp., 11.0 µm; d–j, starches from *Musa*; d, e and g, similar to hybrid type, range, 25.1–55.6 µm; f and h, under polarized light. i, cf. *M. acuminata*, range, 11.7–18.0 µm; j, under polarized light; k, cf. *Nelumbo nucifera*, range, 28.2–31.8 µm; l, *Sagittarria* sp., range, 14.2–21.8 µm; m, cf. *Eleocharis dulcis*, 8.8–12.7 µm; n and o, compound starch grains from *Angiopteris* spp. under brightfield and polarized light, respectively, range, 12.1–32.4 µm; p, *Coix* spp., range, 9.4–22.5 µm. Scale bar, 20 µm.

**Figure 4:**
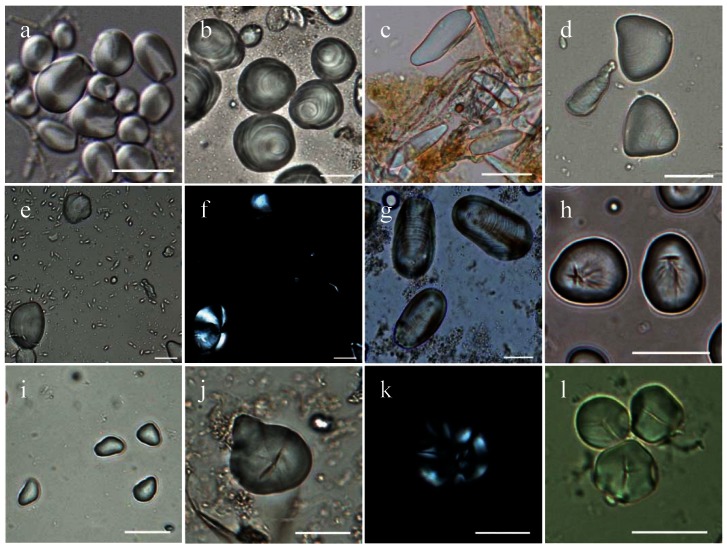
Characteristic starch granules from modern plants. a, *Caryota mitis*, range, 3.3–10.7 µm; b, *Caryota urens*, range, 6.6–45.7 µm; c, *Corypha umbraculifera*, range, 11.4–48.7 µm; d, *Arenga undulatifolia*, range, 3.0–17.8 µm; e–f, *Musa acuminata*, under brightfield and polarized light, range, 6.2–41.3 µm; g, *Nelumbo nucifera* (root), range, 8.0–72.4 µm; h, *Sagittaria trifolia*, range, 9.7–27.6 µm; i, *Eleocharis dulcis*, range, 4.7–18.7 µm; j–k, compound starch grains from *Angiopteris yunnanensis* under brightfield and polarized light, respectively, range, 9.3–149.4 µm; l, *Coix lacryma-jobi*, range, 5.4–20.4 µm. Scale bar, 20 µm except a and c, 10 µm.

A total of 85 starch granules from palms were recovered from seven tools from three ancient living surface including three genera. Some 78 of the 85 starch granules from fishtail palm (*Caryota* sp.) were recovered from six stone tools (Table 2). These granules, ranging 12.1–25.8 µm in size, are typically sub-ovate wide shouldered at the hilum, tapering toward the distal end that sometimes contains a conical concavity with a wide or narrow internal channel running from the distal end to the hilum (Figure 3a). Our reference material includes the species of Burmese fishtail palm (*C. mitis*) (Figure 4a), solitary fishtail palm (*C. urens*) (Figure 4b) and Philippines fishtail palm (*C. cumingii*), and there are strong morphological similarities to the first two, though size is closest to solitary fishtail palm. Six starch granules, ranging12.1–25.8 µm in size (Figure 3b), recovered from six stone tools are identical with modern reference talipot palm (*Corypha umbraculifera*) (Figure 4c). These typically have a sub-spheroid form with characteristic overlapping concentric rings that appear as surface features. A single granule (Figure 3c), 11 µm in size, is narrow at the hilum and elongate with a narrow channel or depression running from the hilum to the distal end (Figure 4d). The physical features are a good match for *Arenga undulatifolia* from our reference collection, but the lack of other *Arenga* sp. from our reference material and other archaeological granules necessarily lowers the certainty of this identification.

Seventeen starch granules from banana (*Musa* sp.) were recovered from three tools recovered from ancient living surface one (Table 2). These can be sub-divided into two groups. Seven starch granules (Figure 3d–h), ranging 25.1–55.6 µm in size, with an irregular or triangular ovoid, well defined lamellae and a wrinkled texture, were recovered from two stone tools on the same living surface, and most closely match those found in the hybrid banana type according to Lentfer [12]. Ten starch granules with a sub-circular to sub-triangular shape (Figure 3i–j), 11.7–18 µm in size, similar to the starches from one of domesticated banana's progenitors, wild seeded banana (*M. acuminata*) (Figure 4e–f).

In total of 48 starch granules from several freshwater roots and tubers were also recovered. Two granules from lotus root (cf. *Nelumbo nucifera*), 31.8 µm and 28.2 µm in length respectively, are distinct elongate ovate with well-defined lamellae and a highly eccentric hilum in the form of a small vacuole (Figure 3k and 4g). Seventeen granules from Chinese arrowhead (*Sagittaria* sp.), ranging 14.2–18 µm in size, are sub-ovate with a centric or slightly offset hilum that is often stellate (Figure 3l and 4h). Twenty-nine granules from water chestnut (cf. *Eleocharis dulcis*) are small (ranging 8.8–12 µm in size), in triangular, oval or irregular shape, sometimes with light fissures through the centric hila (Figure 3m, 4i). From tool #8 (Figure 2) 38 starch granules from the terrestrial fern (*Angiopteris* sp.) were recovered (Figure 3n–o), which includes edible varieties such as *Angiopteris yunnanensis* [19]. Starch granules from this species are typically irregular compound granules with complex extinction crosses under cross polarised light (Figure 4j–k).

**Figure 5:**
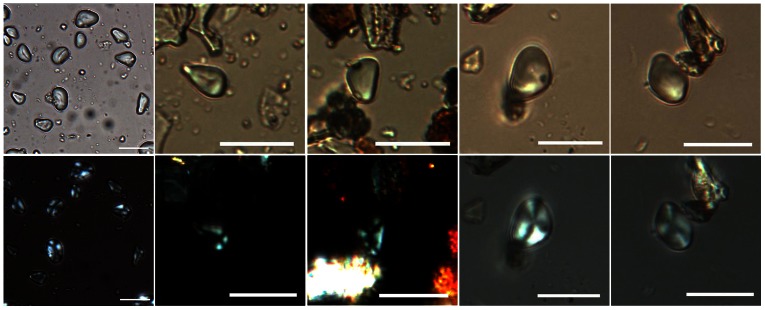
Modern and archaeological starch granules from acorns. The Upper image of each pair shows the starch under brightfield light, and the Lower image shows it under cross-polarized light. The first pair is modern acorn starches (*Quercus acutissima*), range, 5.1–23.7 µm; the remaining four pairs are ancient starches identified as *Quercus* sp., range, 11.8–16.4 µm.

Job's-tear (*Coix* spp.) also seems to have been a common resource exploited at Xincun (Figure 3p). Fifty three granules of Job's-tear were recovered, typically spheroid or polyhedral, with two to three flat facets, centric hilum that normally has a T-shaped fissure (Figure 4l). Five starch granules from acorns (*Quercus* sp.) were also identified (Figure 5).

**Figure 6:**
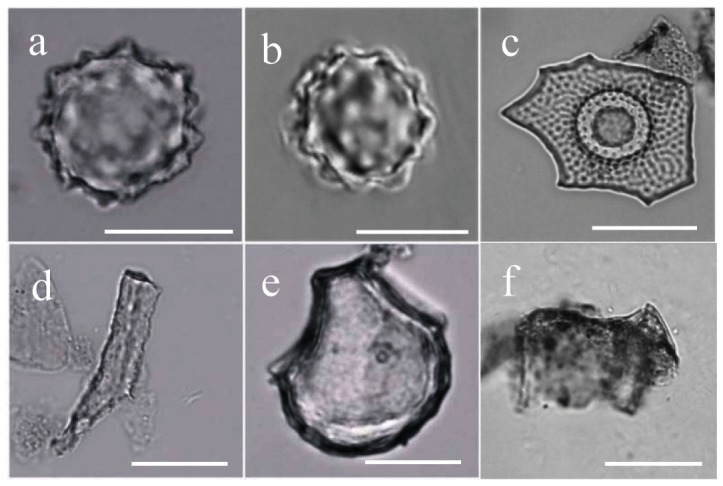
Some phytolith types extracted from tools 9#–12#. a and b, globular echinate (or ‘spherical crenate’) from Aracaceae; e, scale decorated bulliform from *Oryza*; f, glume with two peaks from *Oryza*. Scale bar, 20 µm.

**Figure 7:**
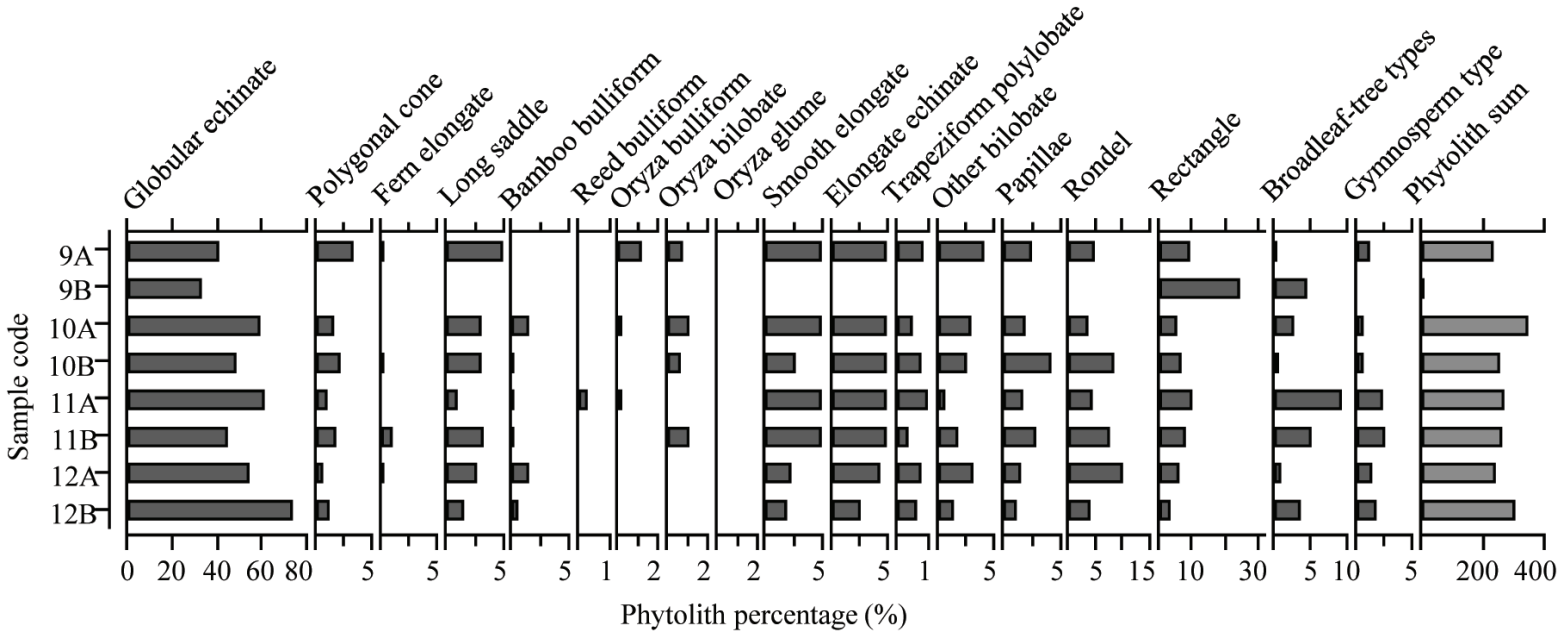
Percentage diagram for selected phytolith types from surface residues of stone tools 9#–12#: A and B indicate the used facet and non-used facet, respectively.

Phytoliths extracted from residues adhering to the stone tools coincide with the starch results at the family level (Figure 6), except for the presence of rice (*Oryza*) phytoliths. Of the identified 1,950 phytoliths, 56% were globular echinate phytoliths from palm (Aracaceae) (Figure 6a–b and Figure 7). Also recorded were polygonal cones from sedge (Cyperaceae) (Figure 6c), which includes water chestnuts, and elongate triangular prisms from ferns (Figure 6d). About one percent of the phytoliths were identified as belonging to the rice (*Oryza*) among which some fan-shaped, two-peak glumes and scale decorated bulliforms were recovered (Figure 6e–g and Figure 7).

## Discussion

Starch and phytolith remains reveal that palm was the dominant exploited plant, reflecting the importance of palms as a resource at Xincun. All palm species identified from starch granules are indigenous to southern subtropical and tropical China or India [20]. Importantly, all three species may be processed for their starchy pith; the product known colloquially as sago or sago flour [21]. The fishtail palms (*Caryota* sp.) are mentioned by Ki Han during the Jin Dynasty (A.D. 290–307) and a starch producing palm is mentioned during the fifth century A.D. as an important agricultural plant [21]. Sugar palm (*Arenga pinnata*), is also known from literary records as a starch resource extracted by the Hakka of southern Hainan Province [22]. In the swampy lowlands of Arunachal Pradesh, in eastern India, fishtail palms and talipot palms were processed for starch [20]. The talipot palm (*Corypha umbraculifera*), occurs in low-lying areas, often near the coast, while fishtail palms (*Caryota* sp.) and gomuti palm (*Arenga* sp.) occur in the lowlands and disturbed rainforest environments; all are frequently found in association with human occupations [20]. Sago palms represent high yields of reliable, digestible carbohydrates, but the food processing is labour intensive [23]–[25]. Sedentary communities of sago-eaters settle near stands of swamp sago (*Metroxylon sagu*), and are likely to have increased the density and size of natural stands by planting suckers in the past [25]–[28]. While it is possible that the occupants of Xincun harvested the stands of wild sago palms growing not far from the site, it is more likely that these palms were managed near the site.

Two progenitors of the modern domesticated banana (*Musa acuminata* and *M. balbisiana*), were also recorded in this region [29]. As with all types of banana, the plant produces a wide range of raw materials including leaves for thatching, rain capes, fibres, edible shoots as well as edible starchy pith [30]. Wild seeded banana (*M. balbisiana*), though not producing edible fruits, appears to have been a plant of interest to people in the Holocene as it was translocated from southern China into island Southeast Asia [31]. The earliest known cultivated bananas (*M. acuminata*), were found in New Guinea highlands at the site of Kuk swamp dating at 6,950 to 6,440 years ago [32]. Early cultivators may not have been interested in the fruit but rather preferred the starch from stem pith and its other ancillary uses [33]–[34]. The presence of banana starches including a hybrid at Xincun site around 5,000 years ago indicates the exploitation of this plant use and probably its local cultivation. Since both two progenitors were recorded in the region, here we cannot rule out the possibility of wild hybrid.

Archaeological starches and phytoliths from the Xincun site show at least four types of granules from roots and tubers, of which water chestnuts, Chinese arrowheads and lotus are still very popular in south China today. Several species of ferns (*Angiopteris* sp.) were widely used as one of the famine foods during 1959–1961known as the Three-year Hardest Time in China when many people passed away due to continuous famines [19]. At that time people were encouraged to find the fern roots in the mountainous areas of south China, but possibly this last extensive and intensive exploitation resulted in the sever danger of extinction of these plants. No starch grains from taro were recovered. One reason is that the taro granules are too small to detect under the microscope and no phytoliths are produced by this plant. The other reason is that the taro was not be used at the time.

From the southern region of Fujian, the earliest rice remains are dated to 2,870–2,340 aBC [35] and from Shixia site in northern Guandong Province, deposits containing rice grains and stalks ranges from 3,000–2,500 aBC [35]. The rice farming had not arrived at the Xincun site in the far southern Guangdong by 5,000 years ago. These dates broadly accord with the known spread of rice into mainland Southeast Asia, which occurs around 2,000 aBC [36]. Direct evidence of rice across Island Southeast Asia, is also poor, often derived from husk impressions or husk inclusions in pottery only [36], [37]. Current archaeobotanical evidence of mid Holocene plant use in the region reveals use of starch rich plants such as yams, aroids, palms and many species of nuts; rice is often absent at these sites [37], [38], [39]. It seems reasonable to consider that the agricultural package in these regions may have relied heavily on tubers, nuts, fruits and palms and there is the possibility that this even precedes the later introduction of rice and rice agriculture [37], [23].

Palms, bananas and root crops are normally propagated vegetatively in these regions. The availability of these wild starchy plants would have made subsistence relatively easy in the region [37]. As well, these plants are not dependent upon year-round human labour, which may have contributed to resistance to more labour intensive rice, until either demography or cultural pressures or other reasons encouraged the labour investment needed in cultivating rice [36].

## Conclusions

In sum, the starch assemblage recovered from the Xincun site incorporated a wide range of edible plants such as sago palms, banana, water chestnuts, lotus roots, arrowheads, ferns, Job's-tears, acorns. The phytolith assemblage indicates the presence of sago palms, sedge, bamboo, ferns with a minute quantity of rice. The dominant starches and phytoliths from palms suggest that the sago palms were an important plant food prior to the rice in south subtropical China. This suite of edible plants provides the first evidence for the exploitation of resources in a coastal village community in southern subtropical China around 5,000 years ago, and may represent a common strategy that prevailed in Southeast Asia before rice farming was practised widely. Because of their reliance on a wide range of starch-rich plant foods, the transition towards labour intensive rice agriculture was a slow process.
